# Efficacy and Safety of Human Epidermal Growth Factor Receptor 2 (HER2) Antibody–Drug Conjugates in Solid Tumors Harboring Non-tyrosine Kinase Domain ERBB2 Mutations: A Systematic Review

**DOI:** 10.7759/cureus.103439

**Published:** 2026-02-11

**Authors:** Ryuichi Ohta, Taichi Fujimori, Kaoru Tanaka, Hidetoshi Hayashi

**Affiliations:** 1 Department of Community Care, Unnan City Hospital, Unnan, JPN; 2 Department of Internal Medicine, Shimane University, Shimane, JPN; 3 Department of Medical Oncology, Kindai University Faculty of Medicine, Sakai, JPN

**Keywords:** erbb2 mutation, her2 antibody–drug conjugate, interstitial lung disease, non–tyrosine kinase domain mutation, solid tumors, trastuzumab deruxtecan

## Abstract

ERBB2 (HER2) gene mutations are established oncogenic drivers across a wide range of solid tumors. While therapeutic development has primarily focused on alterations within the tyrosine kinase domain (TKD), ERBB2 mutations also occur in non-TKD regions, including the extracellular, transmembrane, juxtamembrane, and C-terminal domains. The clinical efficacy and safety of HER2 antibody-drug conjugates (ADCs) in tumors harboring non-TKD ERBB2 mutations remain incompletely characterized. We conducted a systematic review in accordance with the PRISMA 2020 guidelines. PubMed, Embase, and Web of Science were searched for clinical studies reporting outcomes of HER2 ADC therapy in adult patients with solid tumors harboring non-TKD ERBB2 mutations. Eligible studies included phase II non-randomized trials, observational studies, case series, and case reports. Data were extracted on tumor type, mutation domain, ADC regimen, efficacy outcomes, and safety, with particular attention to interstitial lung disease (ILD)/pneumonitis. Nine studies were included, comprising five phase II non-randomized clinical trials and four case-based studies. Non-TKD ERBB2 mutations were most frequently located in the extracellular domain (56 reported cases), followed by the transmembrane/juxtamembrane domains (26 cases), while C-terminal alterations (15 cases) were less commonly reported. Trastuzumab deruxtecan was the most frequently administered ADC and demonstrated objective tumor responses across multiple tumor types, with the most consistent activity observed in tumors harboring extracellular and transmembrane/juxtamembrane domain mutations. In contrast, evidence supporting efficacy in C-terminal domain mutations was limited and derived mainly from case-based reports. ILD/pneumonitis was reported in a subset of patients, including grade ≥3 events, but no ILD-related fatal events were reported among patients with non-TKD ERBB2 mutations. Current clinical evidence indicates that HER2 ADCs can provide meaningful antitumor activity in selected solid tumors harboring non-TKD ERBB2 mutations, particularly those involving the extracellular and transmembrane/juxtamembrane domains. However, treatment benefit appears heterogeneous across mutation domains and less consistent than that reported for canonical TKD alterations, and interpretation is limited by study heterogeneity and the lack of mutation-domain-stratified outcome reporting. Prospective studies with mutation-domain-stratified designs are warranted to better define the role of HER2 ADCs in non-TKD ERBB2-mutated malignancies and to inform precision treatment strategies.

## Introduction and background

Human epidermal growth factor receptor 2 (HER2), encoded by the ERBB2 gene, is a well-established oncogenic driver in a variety of solid tumors, including breast, gastric, and lung cancers [1]. In addition to conventional HER2 gene amplification and protein overexpression, advances in next-generation sequencing technologies have enabled the identification of diverse ERBB2 gene mutations across multiple tumor types [2]. In lung cancer, HER2-mutated adenocarcinoma has been reported to account for approximately 2-4% of cases and has attracted increasing clinical attention as a potential target for molecularly guided therapy [3].

Among ERBB2 mutations, alterations located within the tyrosine kinase domain (TKD), particularly exon 20 insertion mutations, have been the most extensively investigated [4]. Because the TKD plays a central role in intracellular signal transduction, these mutations have served as the primary focus of clinical trials evaluating HER2-targeted therapies [5]. In contrast, ERBB2 mutations are not restricted to the TKD and have been identified in a variety of non-TKD regions, including the extracellular domain, transmembrane domain, juxtamembrane region, and C-terminal tail [6]. However, despite the biological diversity of these non-TKD mutations, their clinical relevance and therapeutic implications remain poorly characterized.

More recently, the development of antibody-drug conjugates (ADCs) has expanded the therapeutic landscape of HER2-targeted treatment. Unlike tyrosine kinase inhibitors, ADCs exert antitumor effects through HER2 binding, receptor internalization, and intracellular delivery of cytotoxic payloads [7]. As this mechanism of action may depend more on HER2 protein expression and internalization rather than direct kinase activation, HER2 ADCs are theoretically expected to be effective even in tumors harboring non-TKD ERBB2 mutations [8]. Trastuzumab deruxtecan (T-DXd) and other HER2-directed ADCs have demonstrated promising clinical activity, and emerging reports suggest potential efficacy in selected patients with non-TKD ERBB2 alterations [9].

Nevertheless, current evidence regarding the efficacy and safety of HER2 ADCs in non-TKD ERBB2-mutated solid tumors is limited. Available data are primarily derived from small clinical studies, subgroup analyses of basket trials, and individual case reports, including only a small number of patients from larger trials such as DESTINY-Lung02 [10]. To date, no comprehensive, systematic evaluation has explicitly focused on non-TKD ERBB2 mutations. As a result, substantial knowledge gaps remain regarding treatment response, safety profiles, and potential differences in clinical outcomes according to mutation location.

Given these limitations, a systematic synthesis of existing clinical evidence is warranted. Therefore, this study aimed to systematically review the efficacy and safety of HER2 ADCs in adult patients with solid tumors harboring non-TKD ERBB2 mutations. By organizing available data according to mutation domain and treatment outcomes, this review seeks to address a critical unmet need in precision oncology and to provide clinically relevant insights to guide future therapeutic strategies [1].

## Review

Methods

Study Design

This study was conducted as a systematic review to evaluate the efficacy and safety of HER2 ADCs in adult patients with solid tumors harboring non-TKD ERBB2 mutations. The review was designed and reported in accordance with the Preferred Reporting Items for Systematic Reviews and Meta-Analyses (PRISMA) 2020 statement [11]. Given the rarity of non-TKD ERBB2 mutations and the limited availability of large-scale clinical trials, a broad range of study designs, including clinical trials, observational studies, case series, and case reports, were considered eligible to ensure comprehensive evidence synthesis.

Protocol Registration

The review protocol was prospectively registered in the International Prospective Register of Systematic Reviews (PROSPERO). The protocol specified the review objectives, eligibility criteria, search strategy, outcomes of interest, and planned methods for data extraction and synthesis. Registration was performed prior to study selection to enhance transparency and methodological rigor. The registered number was CRD420251248812.

Search Strategy

A comprehensive literature search was conducted using PubMed, Embase, and Web of Science. The search strategy was developed to identify clinical studies reporting outcomes of HER2 ADCs in solid tumors with non-TKD ERBB2 mutations. Search terms included combinations of keywords and controlled vocabulary related to ERBB2 or HER2, gene mutations, non-TKD regions (e.g., extracellular, transmembrane, juxtamembrane, and C-terminal domains), and HER2 ADCs such as trastuzumab deruxtecan, trastuzumab emtansine, and other investigational agents. The search was limited to studies published from January 2000 to December 2025 and restricted to articles written in English. Reference lists of relevant articles were manually screened to identify additional eligible studies. The full search strategies for each database are provided in the Appendix to ensure reproducibility.

Study Selection

All retrieved records were imported into a reference management system, and duplicate records were removed prior to screening. Two reviewers independently screened titles and abstracts to assess eligibility according to predefined inclusion and exclusion criteria. Studies were eligible if they included adult patients with solid tumors harboring non-TKD ERBB2 mutations and reported clinical outcomes following treatment with HER2 ADCs. Full-text articles were subsequently reviewed for studies deemed potentially eligible. Discrepancies between reviewers at any stage of the selection process were resolved through discussion and, when necessary, consultation with a third reviewer. Reasons for exclusion at the full-text screening stage were documented. The study selection process is summarized using a PRISMA flow diagram.

Data Extraction and Synthesis

Data extraction was performed independently by two reviewers using a standardized data extraction form. Extracted variables included study characteristics (author, year, country, study design), patient demographics, tumor type, details of ERBB2 mutation location and type, ADC regimen, treatment exposure, efficacy outcomes (objective response rate, disease control rate, progression-free survival, overall survival, and duration of response when available), and safety outcomes, with particular attention to interstitial lung disease (ILD) or pneumonitis. Given the heterogeneity of study designs, tumor types, mutation domains, and outcome reporting, data were synthesized primarily using a narrative approach. Results were organized by the ERBB2 mutation domain and ADC type to highlight potential patterns of clinical response and safety signals.

Statistical Analysis

Formal quantitative meta-analysis was planned only if sufficient homogeneous data were available. For outcomes reported across multiple studies with comparable definitions, pooled estimates of proportions were to be calculated using random-effects models to account for between-study heterogeneity. Statistical heterogeneity was assessed using the I² statistic. When quantitative synthesis was not feasible due to limited sample size or substantial heterogeneity, results were summarized descriptively. Safety outcomes, particularly ILD/pneumonitis, were analyzed qualitatively. All statistical analyses, when performed, were conducted using R software. The methodological quality of included studies was assessed according to study design, using the Risk Of Bias In Non-randomized Studies of Interventions (ROBINS-I) tool, while case series and individual case reports were assessed using the Joanna Briggs Institute (JBI) critical appraisal checklists for case-based studies [12,13]. All statistical analyses were performed using the latest version of EZR (Saitama Medical Center, Jichi Medical University, Saitama, Japan), a graphical user interface for R and R Commander that provides an integrated environment for conducting medical statistics and meta-analyses [14].

Results

Study Selection

The study selection process followed the PRISMA 2020 guidelines and is summarized in the PRISMA flow diagram. A total of 974 records were identified through database searches, including Web of Science (n = 514), PubMed (n = 396), and Embase (n = 64). After removal of duplicates (n = 206; 205 identified by Covidence and one removed manually), 768 records remained for title and abstract screening. During the screening phase, 716 records were excluded based on titles and abstracts. The remaining 52 articles were sought for full-text retrieval and subsequently assessed for eligibility. All 52 full-text articles were successfully retrieved. Of these, 43 studies were excluded after full-text review. The primary reasons for exclusion were non-original articles such as reviews or commentaries (n = 12) and studies involving an ineligible patient population, including those without confirmed non-TKD ERBB2 mutations or those focusing exclusively on TKD alterations (n = 31). Ultimately, nine studies met all predefined inclusion criteria and were included in the final qualitative synthesis. No studies were classified as ongoing, and no studies were awaiting classification at the time of review (Figure 1).

Study Characteristics

A total of nine studies were included in the final qualitative synthesis, comprising 80 adult patients with solid tumors harboring non-TKD ERBB2 mutations. The characteristics of the included studies are summarized in Table 1.

**Table 1:** Characteristics of Included Studies Summary of study design, tumor types, mutation locations, and HER2 antibody–drug conjugates used in the included studies. ADC, antibody–drug conjugate; ERBB2, erb-b2 receptor tyrosine kinase 2; HER2, human epidermal growth factor receptor 2; non-TKD, non–tyrosine kinase domain; NSCLC, non-small cell lung cancer; T-DXd, trastuzumab deruxtecan; T-DM1, ado-trastuzumab emtansine; TMD, transmembrane domain

First author	Year	Study design	Patient's number	Tumor type	Mutation location	Specific mutation	ADC treatments
Li [15]	2018	Single-center, investigator-initiated phase II basket trial Simon two-stage optimal design	3	NSCLC	Extracellular domain Transmembrane domain	S310F, V659, S335C	T-DM1
Li [16]	2022	Phase II, multicenter, single-arm clinical trial	6	NSCLC	Extracellular domain	S310P, S310T	T-DXd
Goto [17]	2023	Randomized, multicenter, blinded, phase II trial	2	NSCLC	Extracellular domain	S310F	T-DXd
Thavaneswaran [18]	2024	Phase II, single-arm, pan-cancer basket trial	5	Lung, Gallbladder, Salivary gland, Bladder, Uterus	Extracellular domain/Juxtamembrane	S310Y/F, R217H, L755S, R678Q, G660D	T-DM1
Li [19]	2024	International, open-label, phase II basket study	51	Multiple solid tumours	Extracellular domain Transmembrane / juxtamembrane domain C-terminal region	S310F, S310Y, R678Q, T862A, V842I	T-DXd
Meng [20]	2025	Case series	4	NSCLC	Transmembrane / juxtamembrane region C-terminal region Extracellular domain	V659E, H878Y, T126A	T-DXd
Li [21]	2025	Multicentre, single-arm, phase II trial	5	NSCLC	Extracellular domain, C-terminal region, Others	S310F, L755P, L869R / T862A	SHR-A1811
Choudhury [22]	2025	Case series	3	Endometrial carcinoma Serous ovarian carcinoma Cervical adenocarcinoma	Kinase domain point mutation Non-canonical deletion affecting extracellular/juxtamembrane region	G776V, HER2 exon 16 deletion, I767M	T-DXd
Nishihara [23]	2025	Single case report	1	NSCLC	Transmembrane domain (TMD)	V659E	T-DXd

Regarding study design, the evidence base consisted predominantly of early-phase, non-comparative studies. Four studies were prospective phase II clinical trials with basket or pan-tumor designs, while the remaining five were case series or individual case reports. No randomized controlled trials specifically designed to evaluate HER2 ADCs in non-TKD ERBB2-mutated tumors were identified. The studies were published between 2018 and 2025, reflecting the rapid evolution of clinical interest in HER2-directed antibody-drug conjugates for molecularly selected patient populations.

The study populations encompassed a broad spectrum of solid tumors, with non-small cell lung cancer (NSCLC) representing the most frequently reported tumor type. Additional malignancies included gallbladder cancer, salivary gland carcinoma, bladder cancer, uterine cancer, endometrial carcinoma, ovarian carcinoma, and cervical adenocarcinoma, underscoring the tumor-agnostic distribution of non-TKD ERBB2 mutations. Across all studies, the presence and location of ERBB2 mutations were explicitly confirmed using molecular diagnostic techniques, most commonly next-generation sequencing.

Regarding mutation location, non-TKD ERBB2 alterations were predominantly identified in the extracellular domain, followed by the transmembrane and juxtamembrane regions. C-terminal domain mutations were infrequently reported, and only a small number of patients with such alterations were included across the dataset. Several studies enrolled patients with heterogeneous non-TKD mutation domains, whereas others focused on specific mutation sites, most notably S310 substitutions in the extracellular domain and V659E in the transmembrane domain.

All patients received HER2-directed antibody-drug conjugate therapy. T-DXd was the most frequently administered agent and accounted for the majority of treated cases. Other HER2 ADCs, including ado-trastuzumab emtansine (T-DM1) and the investigational agent SHR-A1811 (Trastuzumab rezetecan), were reported less commonly. Treatment exposure varied across studies, reflecting differences in tumor type, prior lines of therapy, and study design.

Efficacy outcomes were reported heterogeneously. Objective response rate (ORR) or individual response categories were available in most studies, whereas progression-free survival, overall survival, and duration of response were inconsistently reported and often limited to descriptive case-level data. Safety outcomes, including adverse events related to HER2 ADC therapy, were described in all included studies, with particular attention to ILD or pneumonitis, a recognized toxicity of HER2-directed ADCs.

Mutation Domain-Specific Efficacy

The efficacy of HER2 ADCs appeared to vary according to the location of ERBB2 mutations outside the tyrosine kinase domain (non-TKD). However, interpretation was limited by heterogeneity in mutation distribution, tumor types, and study design. Across the included studies, non-TKD ERBB2 mutations were most frequently identified in the extracellular domain, followed by the transmembrane and juxtamembrane regions, whereas C-terminal alterations were infrequently reported. Due to the rarity of non-TKD ERBB2 mutations and the predominance of basket trials and case-based reports, efficacy outcomes were synthesized descriptively and are summarized according to mutation domain in Table 2.

**Table 2:** Summary of Efficacy According to the ERBB2 Mutation Domain Distribution of non–tyrosine kinase domain ERBB2 mutation domains among included patients. Counts may overlap due to multiple mutations in individual patients. ADC, antibody–drug conjugate; ERBB2, erb-b2 receptor tyrosine kinase 2; HER2, human epidermal growth factor receptor 2.

ERBB2 mutation domain (non-TKD)	Reported cases (overlapping)	Notes
Extracellular domain	56	Includes S310F/Y/P/T, exon 16 deletion; overlapping counts allowed
Transmembrane/Juxtamembrane domain	26	Includes V659E, R678Q, exon 16 deletion; overlapping counts allowed
C-terminal domain	15	Includes T862A, V842I, H878Y, L869R; overlapping counts allowed

Extracellular Domain Mutations

Extracellular domain mutations constituted the most frequently reported non-TKD ERBB2 alterations across the included studies. When aggregated across the dataset, 56 reported cases involved extracellular domain mutations, most commonly substitutions at codon S310 (S310F, S310Y, S310P, and S310T), as well as non-TKD structural alterations such as exon 16 deletions.

In the DESTINY-PanTumor01 phase II basket study, 34 of 102 patients (33%) harbored extracellular domain mutations, predominantly S310F (n = 27) and S310Y (n = 7), and objective tumor responses were observed across multiple tumor types within this subgroup. These responses contributed to the overall confirmed ORR of 29.4% (30 of 102 patients) reported for the full study population, although response rates were not stratified by the mutation domain.

Beyond basket trial data, case reports and small case series documented objective responses (partial or complete responses) to trastuzumab deruxtecan in patients with extracellular domain mutations, including NSCLC and gynecologic malignancies. Taken together, the numerical predominance of reported cases (56 cases) and the recurrent observation of objective responses indicate that extracellular domain alterations are consistently associated with antitumor activity following HER2 ADC therapy and represent the strongest quantitative evidence base among non-TKD mutation domains.

Transmembrane and Juxtamembrane Domain Mutations

Mutations located in the transmembrane or juxtamembrane regions represented the second most frequently reported non-TKD alterations, accounting for 26 reported cases across the included studies. Frequently observed variants included V659E in the transmembrane domain and R678Q in the juxtamembrane region.

Within DESTINY-PanTumor01, 17 of 102 patients (17%) harbored transmembrane or juxtamembrane mutations, and objective responses and durable disease control were observed in selected patients within this subgroup, although domain-specific response rates were not separately reported. Consistent with these findings, case-based studies described radiographic tumor shrinkage and sustained disease stabilization in patients with V659E or R678Q mutations treated with HER2 ADCs.

Despite the limited number of reported cases (26 cases) and heterogeneous outcome reporting, the repeated documentation of objective responses and disease control supports the conclusion that HER2 ADCs retain clinically meaningful antitumor activity in non-TKD mutations located proximal to the cell membrane.

C-terminal Domain Mutations

Evidence regarding the efficacy of HER2 ADCs in tumors harboring C-terminal domain ERBB2 mutations was comparatively sparse. Across the dataset, 15 reported cases involved C-terminal alterations, including T862A (n = 4), V842I (n = 9), H878Y (n = 1), and L869R/T862A (n = 1). These cases were derived almost exclusively from case reports or small case series.

Among these reports, objective tumor responses were infrequently described, and most publications lacked systematic reporting of response depth or duration. Given the small number of reported cases (15), overlapping mutation patterns, and the absence of domain-stratified efficacy outcomes, no quantitative conclusions regarding the clinical benefit of HER2 ADC therapy in C-terminal ERBB2 mutations can be drawn at present.

Summary of Domain-Specific Patterns

Overall, the available evidence indicates heterogeneous antitumor efficacy of HER2 antibody-drug conjugates across non-TKD ERBB2 mutation domains. Quantitatively, the largest body of evidence supports efficacy in extracellular domain mutations (56 reported cases; 34 cases in DESTINY-PanTumor01), followed by transmembrane/juxtamembrane alterations (26 reported cases; 17 cases in DESTINY-PanTumor01). In contrast, evidence for C-terminal domain mutations remains limited (15 reported cases), with objective responses rarely documented.

These domain-specific numerical differences highlight the clinical relevance of mutation location and underscore the need for future prospective studies with domain-stratified efficacy reporting to more precisely define treatment benefit in patients with non-TKD ERBB2-mutated solid tumors (Table 3).

**Table 3:** Domain-Specific Numerical Differences Summary of domain-specific efficacy patterns of HER2 antibody–drug conjugates in non-TKD ERBB2–mutated tumors based on clinical trials and case reports. ADC, antibody–drug conjugate; ERBB2, erb-b2 receptor tyrosine kinase 2; HER2, human epidermal growth factor receptor 2; non-TKD, non–tyrosine kinase domain; ORR, objective response rate.

ERBB2 mutation domain (non-TKD)	Reported cases	Major representative mutations	Quantitative efficacy signals	Evidence source
Extracellular domain	56	S310F (n=27), S310Y (n=7), S310P, S310T, exon 16 deletion	Objective responses reported; 34/102 patients in DESTINY-PanTumor01; contributed to overall ORR 29.4% (30/102)	Phase II basket trials; case reports/series
Transmembrane / Juxtamembrane domain	26	V659E, R678Q	Objective responses and durable disease control reported; 17/102 patients in DESTINY-PanTumor01	Phase II basket trials; case reports/series
C-terminal domain	15	T862A (n=4), V842I (n=9), H878Y (n=1), L869R/T862A (n=1)	Objective responses rarely reported; quantitative efficacy not established	Case reports/series only

Non-small Cell Lung Cancer-Specific Outcomes

NSCLC represented the most frequently reported tumor type among the studies included in this systematic review. Based on case-level extractable data, at least 21 patients with NSCLC harboring non-TKD ERBB2 mutations were identified across six studies.

Among these NSCLC cases, extracellular domain mutations were the most common (≥12 cases), followed by transmembrane or juxtamembrane alterations (≥6 cases), while C-terminal domain mutations were less frequently reported (≥3 cases). The most frequently observed extracellular domain variants involved codon S310 (e.g., S310F, S310Y, and S310P), whereas transmembrane and juxtamembrane mutations included variants such as V659E and R678Q.

Trastuzumab deruxtecan was the predominant HER2 antibody-drug conjugate administered in this subgroup, accounting for at least 18 of the 21 NSCLC cases, while other ADCs, including investigational agents, were used less frequently. Objective tumor responses, defined as partial or complete responses, were reported in at least 10 NSCLC patients. Disease control, defined as partial response or stable disease, was observed in at least 14 cases. Reported responses were most frequently associated with extracellular and transmembrane/juxtamembrane domain mutations. Due to heterogeneous reporting and overlapping study populations, formal response rates could not be calculated, and these findings should be interpreted descriptively.

Safety Outcomes (Interstitial Lung Disease/Pneumonitis)

Safety outcomes were reported across all included studies, with particular attention to ILD or pneumonitis, a recognized adverse event associated with HER2 ADCs, especially trastuzumab deruxtecan. Among patients with non-TKD ERBB2 mutations, ILD/pneumonitis of any grade was reported in a subset of treated patients, although incidence rates were not uniformly quantified across studies.

In the DESTINY-PanTumor01 phase II basket study, ILD/pneumonitis was identified as a treatment-related adverse event, consistent with the established safety profile of trastuzumab deruxtecan. While domain-specific ILD incidence according to ERBB2 mutation location was not reported, ILD events were observed across the study population and were not restricted to a particular non-TKD mutation domain. Importantly, no ILD-related fatal events were reported among patients with non-TKD ERBB2 mutations included in this review.

Across the included studies, most reported ILD/pneumonitis events were low-grade (grade 1-2) and were managed with treatment interruption, corticosteroids, and supportive care. Grade ≥3 ILD/pneumonitis was reported only in a limited number of cases, underscoring the potential for clinically significant pulmonary toxicity but indicating that severe events were uncommon in the available dataset.

Similarly, in the earlier phase II basket trial evaluating ado- T-DM1, pulmonary toxicity was infrequently observed, and no treatment-related deaths due to ILD or pneumonitis were reported. Case reports and small case series included in this review described sporadic ILD/pneumonitis events, with heterogeneous reporting of severity, timing of onset, and management strategies.

Overall, the available evidence suggests that the safety profile of HER2 ADCs in patients with non-TKD ERBB2-mutated solid tumors is broadly consistent with that observed in unselected HER2-altered populations, particularly with respect to ILD/pneumonitis. However, due to limited patient numbers, heterogeneous adverse event reporting, and the absence of mutation domain-stratified safety analyses, the domain-specific risk of ILD in non-TKD ERBB2 mutations could not be determined from the current evidence base.

Quality Assessment Results

The methodological quality of the included studies was assessed according to study design using established appraisal tools. The two phase II basket trials were evaluated using ROBINS-I tool, while case series and individual case reports were assessed using JBI critical appraisal checklists for case-based studies (Table 4).

**Table 4:** Risk of Bias Assessment Risk of bias assessment of included studies using ROBINS-I for clinical trials and JBI checklists for case-based studies. JBI, Joanna Briggs Institute; ROBINS-I, Risk Of Bias In Non-randomized Studies of Interventions.

First author	Study design	Assessment tool	Overall risk of bias	Key considerations
Li [15]	Phase II single-arm basket trial	ROBINS-I	Moderate	Single-arm design; no comparator; prospective enrollment; standardized response assessment
Li [16]	Phase II multicenter single-arm trial	ROBINS-I	Moderate	Single-arm design; small sample size; non-TKD subgroup analysis
Goto [17]	Phase II randomized, dose-blinded trial	ROBINS-I	Moderate	Randomization without mutation-domain stratification; limited non-TKD sample
Thavaneswaran [18]	Phase II single-arm pan-cancer basket trial	ROBINS-I	Moderate	Heterogeneous tumor types; aggregated reporting by mutation domain
Li [19]	International, open-label, phase II basket study	ROBINS-I	Moderate	Case-level extractable data; overlapping reporting with basket trial
Meng [20]	Case series	JBI checklist	Moderate	Detailed clinical course reported; small sample size
Li [21]	Phase II multicenter single-arm trial	ROBINS-I	Moderate	Investigational ADC; limited sample size; descriptive efficacy reporting
Choudhury [22]	Case series	JBI checklist	Low to moderate	Clear mutation confirmation and treatment exposure; limited number of cases
Nishihara [23]	Case report	JBI checklist	Moderate	Clear mutation location and response; limited follow-up

Overall, five phase II non-randomized clinical trials were assessed using ROBINS-I, while four case-based studies (case series or single case reports) were evaluated using the JBI checklists. No randomized controlled trials were identified. All phase II non-randomized trials were judged to have a moderate risk of bias according to ROBINS-I. The principal sources of bias were related to single-arm designs, lack of comparator groups, and heterogeneity in tumor types and mutation domains. Nevertheless, these studies demonstrated important methodological strengths, including prospective enrollment, predefined eligibility criteria, systematic response assessment, and standardized safety reporting, supporting their inclusion in the qualitative synthesis.

The remaining case series and individual case reports exhibited inherent methodological limitations, such as small sample size, retrospective reporting, and limited follow-up. However, all case-based studies clearly documented ERBB2 mutation location, ADC treatment exposure, and clinical outcomes, enabling meaningful qualitative interpretation.

No study was excluded based on quality assessment alone. Instead, the results of the quality assessment were used to contextualize the strength of evidence and to justify a descriptive rather than quantitative synthesis of efficacy and safety outcomes, particularly given the rarity of non-TKD ERBB2 mutations and the heterogeneity of available data.

Discussion

Summary of the Study

In this systematic review, we synthesized the available clinical evidence on the efficacy and safety of HER2 ADCs in adult patients with solid tumors harboring non-TKD ERBB2 mutations. Across nine eligible studies, including five phase II non-randomized clinical trials and four case-based studies, HER2 ADCs, most notably trastuzumab deruxtecan, demonstrated clinically meaningful antitumor activity in a subset of patients with non-TKD ERBB2 alterations.

When examined by mutation domain, treatment responses were observed most consistently in tumors harboring extracellular domain mutations (56 reported cases), followed by transmembrane or juxtamembrane alterations (26 reported cases). In contrast, evidence supporting efficacy in C-terminal domain mutations (15 reported cases) was limited and derived almost exclusively from case reports or small case series. Safety outcomes, particularly ILD or pneumonitis, were broadly consistent with the known toxicity profile of HER2 ADCs, with no ILD-related fatal events reported among patients with non-TKD ERBB2 mutations included in this review.

Comparison with Other Studies

Previous clinical investigations of HER2-targeted therapies have primarily focused on ERBB2 TKD alterations, particularly exon 20 insertions, due to their direct role in kinase activation and oncogenic signaling [24-26]. In contrast, non-TKD ERBB2 mutations have historically been regarded as biologically heterogeneous and of uncertain therapeutic relevance [27,28].

The emergence of HER2 ADCs has challenged this paradigm by providing a mechanism of action that is less dependent on kinase activity and instead relies on HER2 binding, receptor internalization, and intracellular payload delivery [29,30]. Our findings are consistent with reports from pan-tumor basket trials demonstrating antitumor activity of HER2 ADCs across diverse tumor types and mutation subgroups. Notably, extracellular and juxtamembrane domain mutations, particularly substitutions involving codon S310 and R678Q, were repeatedly associated with objective responses in both clinical trials and case-based reports.

By organizing the available evidence according to the mutation domain, rather than aggregating all ERBB2 mutations, this review highlights domain-specific patterns of efficacy that may be obscured in broader analyses. In particular, the numerical predominance of extracellular domain cases and the reproducibility of responses across independent studies support a biologically and clinically meaningful role for HER2 ADCs in selected non-TKD ERBB2-mutated tumors [9].

Although HER2 ADCs demonstrated antitumor activity in selected tumors harboring non-TKD ERBB2 mutations, the magnitude and consistency of treatment benefit appear less robust than those reported for canonical TKD alterations, particularly exon 20 insertions [31]. In pivotal studies focusing on TKD mutations, objective response rates and progression-free survival have been reported with greater consistency and statistical precision. In contrast, evidence supporting efficacy in non-TKD mutations, especially outside the extracellular and juxtamembrane domains, relies largely on subgroup analyses and case-based reports, with limited domain-stratified outcome data [18,19].

From a biological perspective, TKD mutations directly activate HER2 kinase signaling and may be associated with higher receptor activation and internalization efficiency, potentially enhancing ADC-mediated payload delivery [32,33]. Non-TKD mutations, by contrast, represent a heterogeneous group with variable functional consequences, which may translate into more heterogeneous and, in some cases, attenuated clinical responses to HER2 ADC therapy [34]. However, direct comparative analyses between TKD and non-TKD mutations are lacking, and these observations should be interpreted cautiously.

Strengths of the Study

This study has several strengths. First, to our knowledge, it represents the first systematic review specifically focused on HER2 ADC efficacy in non-TKD ERBB2-mutated solid tumors. Second, we employed a transparent and methodologically rigorous approach, including prospective protocol registration, a comprehensive literature search, and structured quality assessment using ROBINS-I and JBI critical appraisal tools tailored to study design. Third, by quantitatively summarizing mutation domain distribution (56 extracellular, 26 transmembrane/juxtamembrane, and 15 C-terminal cases), this review provides clinically actionable insights into the heterogeneity of treatment response within the non-TKD ERBB2 population. Finally, safety outcomes, particularly ILD, were explicitly evaluated, reflecting a key concern in real-world ADC use.

Limitations

Several limitations should be acknowledged. The overall evidence base remains limited in size and heterogeneous, with most data derived from single-arm phase II trials and case-based studies rather than randomized controlled trials. Efficacy and safety outcomes were inconsistently reported, and mutation domain-specific response rates were rarely stratified, precluding quantitative meta-analysis. In addition, overlapping mutation domains within individual studies prevented mutually exclusive patient-level aggregation. The small number of reported cases of the C-terminal domain further limited meaningful evaluation of treatment benefit in this subgroup. These limitations necessitate cautious interpretation of the findings and support a descriptive rather than quantitative synthesis.

## Conclusions

The available clinical evidence indicates that HER2 ADCs can induce meaningful antitumor activity in selected patients with non-TKD ERBB2-mutated solid tumors, particularly those harboring extracellular and transmembrane/juxtamembrane domain mutations. The safety profile of HER2 ADCs in this population appears consistent with that observed in broader HER2-altered cohorts, with no new safety signals identified. However, substantial knowledge gaps remain, especially regarding domain-specific efficacy and toxicity. Prospective studies with mutation-domain-stratified designs and standardized outcome reporting are warranted to better define the therapeutic role of HER2 ADCs in non-TKD ERBB2-mutated malignancies and to inform precision treatment strategies. While HER2 ADCs provide meaningful antitumor activity in selected non-TKD ERBB2-mutated tumors, the available evidence suggests that treatment benefit may be less consistent than that observed in canonical TKD mutations, underscoring the need for prospective, mutation-domain-stratified studies to refine patient selection and optimize therapeutic strategies.

## Appendices

Detailed literature search strategy

Overview

A comprehensive and reproducible literature search was conducted to identify clinical studies reporting the efficacy and/or safety of HER2 antibody-drug conjugates (ADCs) in solid tumors harboring non-tyrosine kinase domain (non-TKD) ERBB2 mutations. The search strategy was developed in accordance with PRISMA 2020 recommendations and the registered PROSPERO protocol.

Searches were performed in PubMed (MEDLINE), Embase, and Web of Science Core Collection, covering publications from January 1, 2000 to December 31, 2025, and were restricted to English-language articles. The strategy combined controlled vocabulary terms (MeSH and Emtree) and free-text keywords related to ERBB2/HER2, gene mutations, non-TKD mutation domains, and HER2-directed ADCs. Reference lists of all eligible articles and relevant reviews were manually screened to identify additional studies.

PubMed (MEDLINE)

Database: PubMed Date range: January 1, 2000 - December 31, 2025 Language: English

( ("ERBB2"[Mesh] OR "Receptor, ErbB-2"[Mesh] OR ERBB2[tiab] OR HER2[tiab] OR "HER-2"[tiab]) AND (mutation*[tiab] OR mutant*[tiab] OR alteration*[tiab] OR variant*[tiab]) AND ( "extracellular domain"[tiab] OR transmembrane[tiab] OR juxtamembrane[tiab] OR "C-terminal"[tiab] OR "non-tyrosine kinase"[tiab] OR "non-TKD"[tiab] OR "exon 16 deletion"[tiab] ) ) AND ( "Antibody-Drug Conjugates"[Mesh] OR "antibody drug conjugate*"[tiab] OR ADC[tiab] OR "trastuzumab deruxtecan"[tiab] OR T-DXd[tiab] OR "trastuzumab emtansine"[tiab] OR T-DM1[tiab] OR "trastuzumab rezetecan"[tiab] OR SHR-A1811[tiab] ) AND ( "Neoplasms"[Mesh] OR cancer*[tiab] OR tumor*[tiab] OR tumour*[tiab] OR carcinoma*[tiab] )

Embase (Elsevier)

Database: Embase Date range: 2000-2025 Language: English Search fields: Title, Abstract, Keywords

( 'erbb2'/exp OR 'her2'/exp OR erbb2:ti,ab OR her2:ti,ab ) AND ( mutation*:ti,ab OR mutant*:ti,ab OR alteration*:ti,ab OR variant*:ti,ab ) AND ( 'extracellular domain':ti,ab OR transmembrane:ti,ab OR juxtamembrane:ti,ab OR 'c terminal':ti,ab OR 'non tyrosine kinase':ti,ab OR 'non tkd':ti,ab OR 'exon 16 deletion':ti,ab ) AND ( 'antibody drug conjugate'/exp OR 'trastuzumab deruxtecan'/exp OR 'trastuzumab emtansine'/exp OR adc:ti,ab OR 't-dxd':ti,ab OR 't-dm1':ti,ab OR 'shr-a1811':ti,ab ) AND ( 'neoplasm'/exp )

Web of Science Core Collection

Database: Web of Science Core Collection Indexes searched: Science Citation Index Expanded (SCI-EXPANDED) Timespan: 2000-2025 Language: English

TS=( (ERBB2 OR HER2) AND (mutation* OR mutant* OR alteration* OR variant*) AND ( "extracellular domain" OR transmembrane OR juxtamembrane OR "C-terminal" OR "non-tyrosine kinase" OR "non-TKD" OR "exon 16 deletion" ) AND ( "antibody drug conjugate" OR ADC OR "trastuzumab deruxtecan" OR "trastuzumab emtansine" OR T-DXd OR T-DM1 OR SHR-A1811 ) AND (cancer* OR tumor* OR tumour* OR carcinoma*) )
